# Supplementary figures and images for: The interplay between Wnt and mTOR signaling modulates ciliogenesis in human retinal epithelial cells
Source: PLoS Biol. 2025 Sep 2;23(9):e3003369. doi: 10.1371/journal.pbio.3003369 (PMC12416842; doi:10.1371/journal.pbio.3003369)

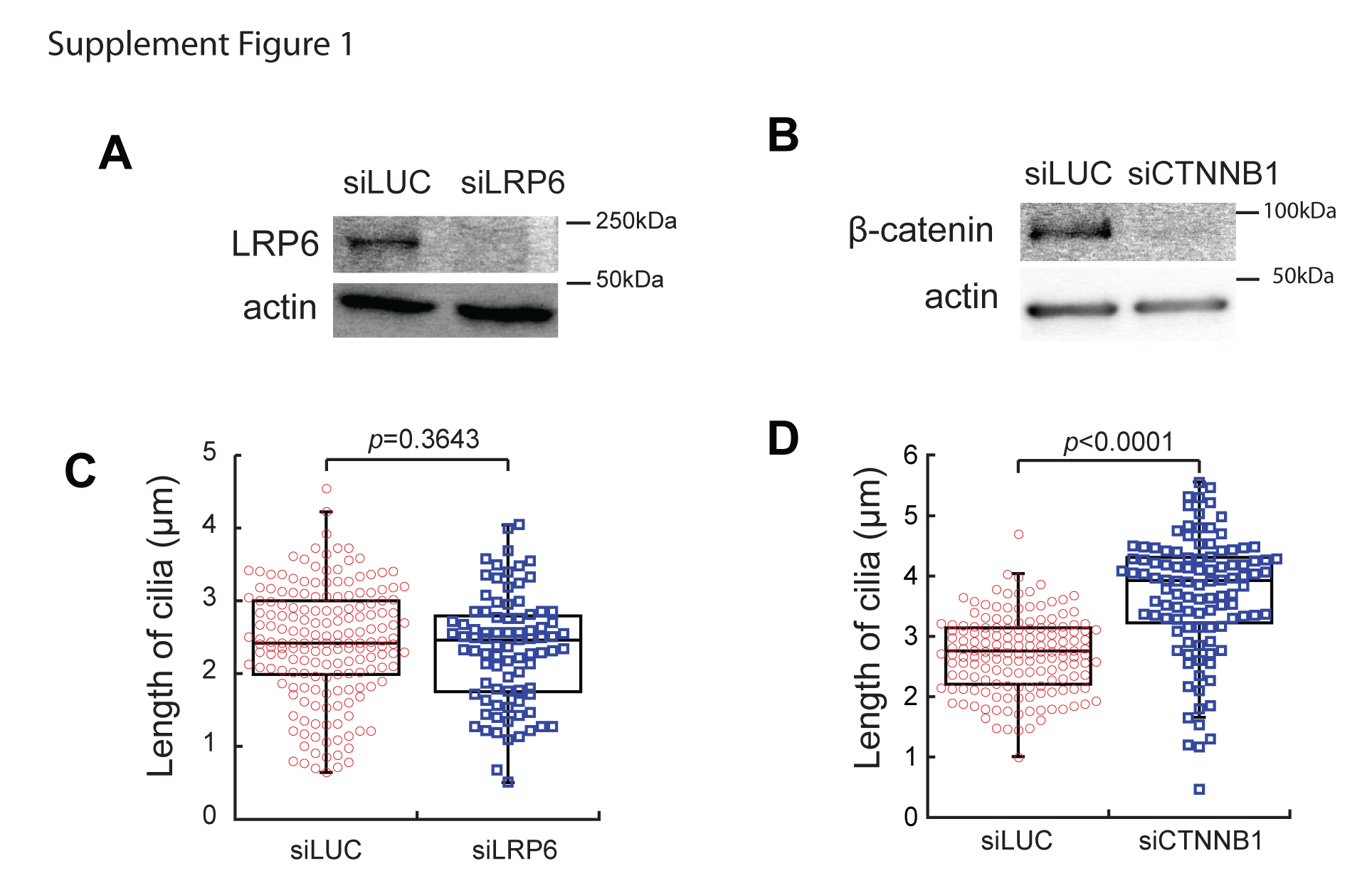

Supplement: S1 Fig — (A) Western blot analysis of LRP6 in control (siLUC) and LRP6-depleted (siLRP6) RPE1 cells, serum starved for 24 h. Actin served as a loading control. (B) Western blot analysis of β-catenin in control (siLUC) and β-catenin-depleted (siCTNNB1) RPE1 cells, serum starved for 24 h. Actin served as a loading control. (C) Quantification of ciliary length from (A). The box/dot plots show quantification of ciliary length from three independent experiments. siLUC, n = 180; siLRP6, n = 87. (D) Quantification of ciliary length from (B). The box/dot plots show quantification of ciliary length from three independent experiments. siLUC, n = 160; siCTNNB1, n = 107. P values are based on Student t test. The data underlying the graphs and blots in this figure can be found in the S2 Data and S1 Raw Images files. (S1_Fig.TIF) [file pbio.3003369.s001.tif]

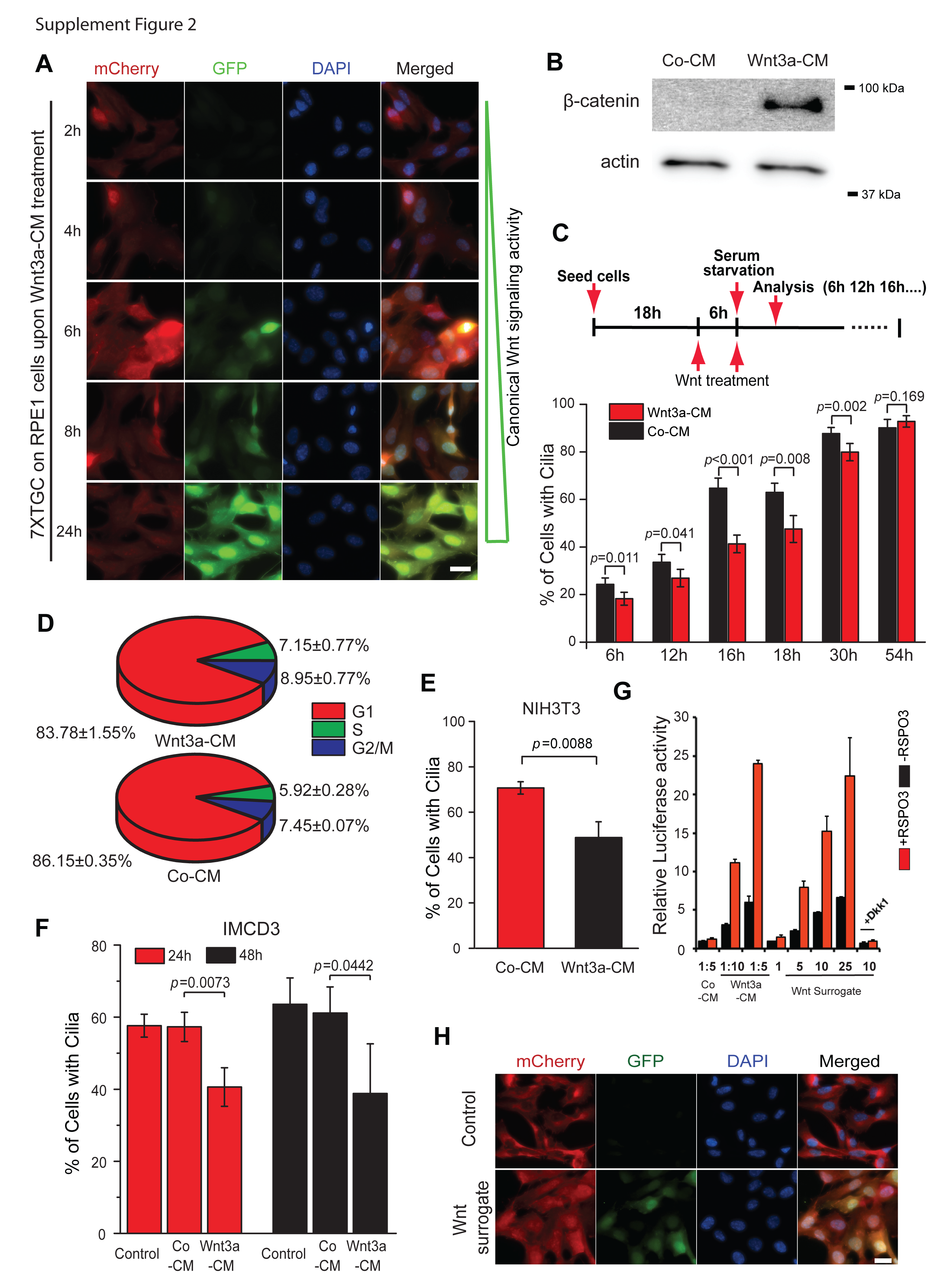

Supplement: S2 Fig — (A) Images of RPE1 cells expressing the 7xTGC Wnt reporter construct treated with Wnt3a-CM for the indicated time points. The increase in GFP signal reflects Wnt activation. (B) Western blot analysis of saponin lysed RPE1 cells treated with Co-CM or Wnt3a-CM (as depicted in Fig 1C) and serum starved for 48 h. Actin served as a loading control. (C) Experimental setup and quantification of ciliation of RPE1 cells treated with Co-CM and Wnt3a-CM at the indicated times after serum starvation. The bar graph indicates the mean ± S.D. from three independent experiments. At least 150 cells were counted per sample and time point. (D) Pie chart showing the percentage of RPE1 cells in G1, S, and G2/M phases of the cell cycle as determined by FACS-based DNA content analysis after Co-CM and Wnt3a-CM treatment (as depicted in C) and 16 h of serum starvation. (E) Quantification of ciliation in NIH3T3 cells treated with Co-CM (n = 461) and Wnt3a-CM (n = 493) and serum starved for 16 h. The bar graph indicates the mean ± S.D. from three independent experiments. (F) Quantification of ciliation in IMCD3 cells treated with Co-CM or Wnt3a-CM and serum starved for 24 and 48 h. The bar graph indicates the mean ± S.D. from three independent experiments. (G) Relative luciferase activity in HEK293T cells treated either with Co-CM or Wnt3a-CM at the indicated dilutions or with purified recombinant surrogate Wnt agonist (concentrations varying between 1 and 10 µg/ml as indicated). Reactions were done in the absence (black bars) or presence (red bars) of R-Spondin to enhance Wnt signaling. DKK1 (Wnt inhibitor) was used as a control. Mean ± S.D. from three independent experiments are shown. (H) Images of RPE1 7xTGC cells treated for 6 h with buffer (control) or 10 µg/ml of surrogate Wnt agonist. The increase in GFP signal reflects Wnt activation. Scale bar: 10 µm. P values are based on Student t test. The data underlying the graphs and blots in this figure can be found in the S2 Data and S1 Raw [file pbio.3003369.s002.tif]

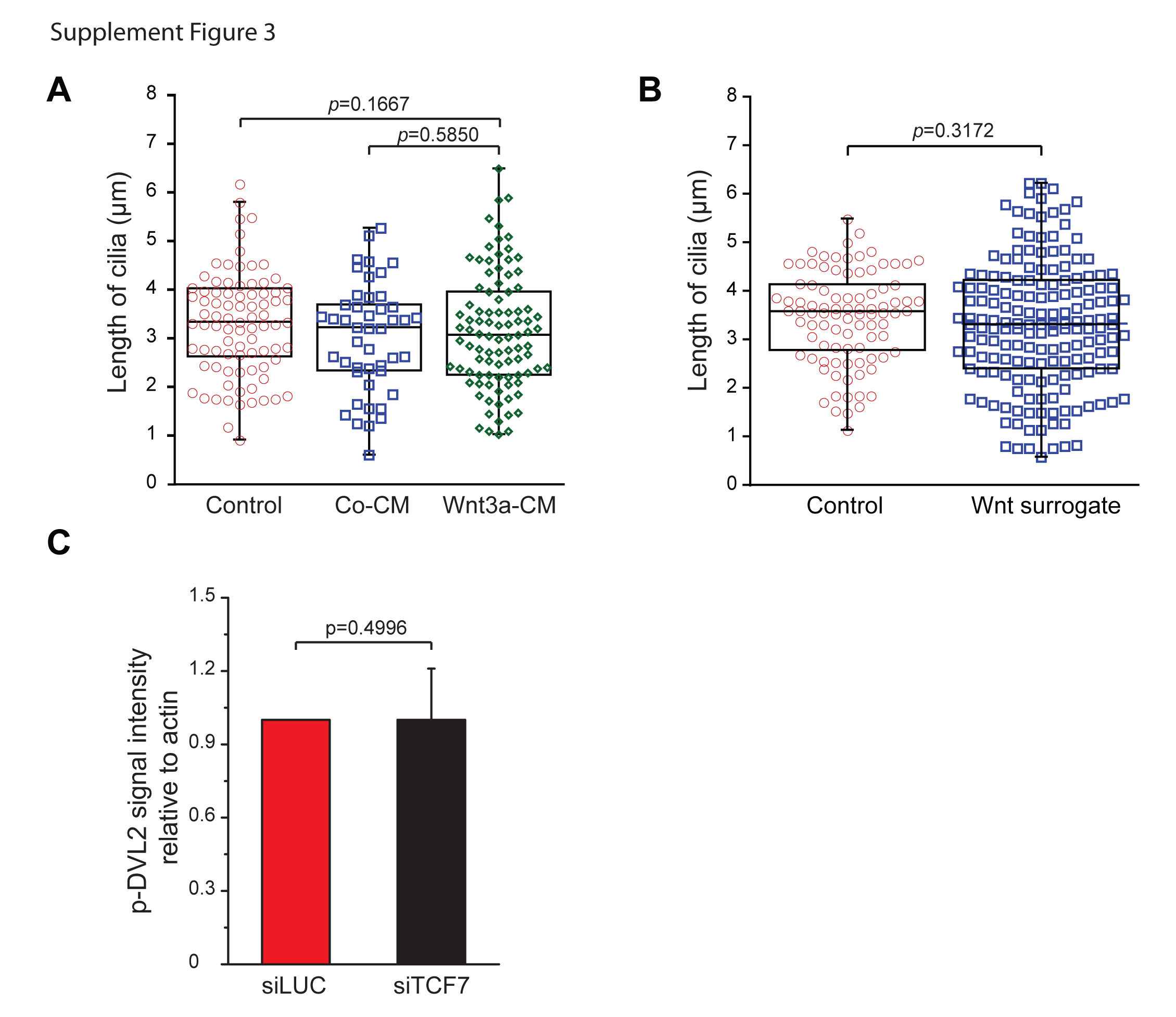

Supplement: S3 Fig — (A) Quantification of ciliary length of RPE1 cells treated with serum-free medium (control), Co-CM and Wnt3a-CM and serum starved for 16 h. The box/dot plots show quantification of ciliary length from three independent experiments. Control, n = 90; Co-CM, n = 95; Wnt3a-CM, n = 47. (B) Quantification of ciliary length of RPE1 cells treated with buffer control (n = 95) or purified surrogate Wnt agonist (n = 175) after 16 h of serum starvation. The box/dot plots show quantification of ciliary length from three independent experiments. (C) Quantification of Fig 2B showing the relative signal intensity of p-DVL2 to actin in siLUC and siTCF7 Wnt3a-CM treated samples. The bar graph indicates the mean ± S.D. of three independent experiments. P values are based on Student t test. The data underlying the graphs in this figure can be found in the S2 Data file. (S3_Fig.TIF) [file pbio.3003369.s003.tif]

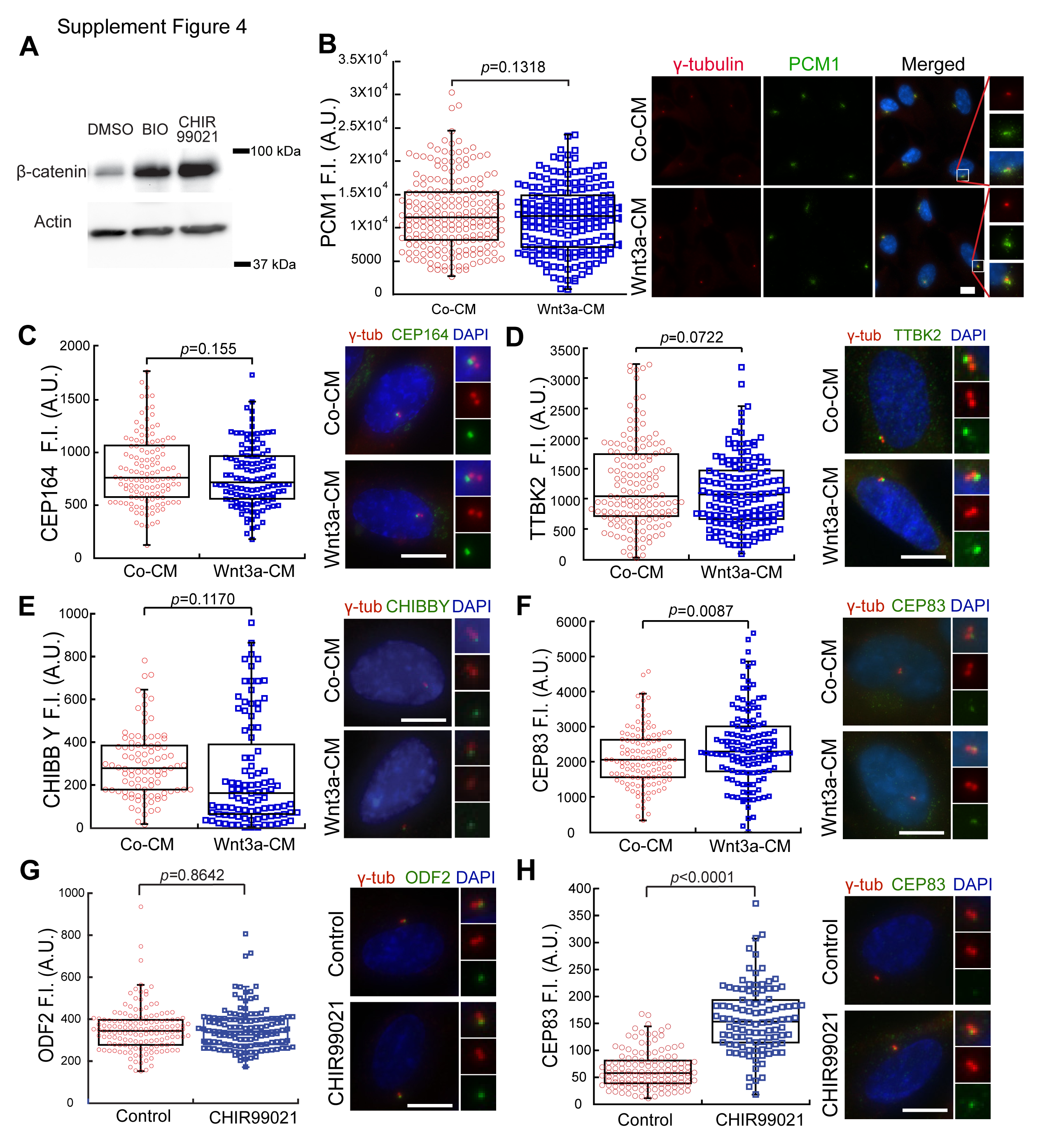

Supplement: S4 Fig — (A) Western blot analysis of saponin lysed RPE1 cells shows the levels of cytoplasmic β-catenin after 16 h of serum starvation and treatment with DMSO, BIO or CHIR99021. Actin served as a loading control. (B) Representative images and quantification of PCM1 fluorescence intensity at the centrosomal area (in arbitrary units) in RPE1 cells treated with Co-CM and Wnt3a-CM as depicted in S2C and serum-starved for 16 h. γ-tubulin (red) and DAPI (blue) served as markers for centrosomes and nuclei, respectively. Magnifications of the centrosomal area are shown on the right as indicated. Co-CM, n = 214; Wnt3a-CM, n = 187. Scale bar: 10 µm. (C–F) RPE1 cells were treated with Co-CM and Wnt3a-CM and serum starved for 16 h as depicted in Fig 1C. Cells were stained for CEP164 (C), TTBK2 (D), CHIBBY (E), and CEP83 (F). γ-tubulin served as a centriolar marker. DNA was stained with DAPI. The graph shows the fluorescence intensity of the corresponding proteins at centrioles from three independent experiments. Representative images with enlargements of the centrosomal area are shown on the right. (C) Co-CM, n = 132; Wnt3a-CM, n = 115; (D) Co-CM, n = 150; Wnt3a-CM, n = 140; (E) Co-CM, n = 103; Wnt3a-CM, n = 112; (F) Co-CM, n = 122; Wnt3a-CM, n = 133. Scale bar: 10 µm. (G, H) RPE1 cells were treated with DMSO and CHIR99021 and serum starved for 16 h as depicted in Fig 1C. Cells were stained for ODF2 (G) and CEP83 (H). γ-tubulin served as a centriolar marker. DNA was stained with DAPI. The graph shows the fluorescence intensity of the corresponding proteins at centrioles from three independent experiments. Representative images with enlargements of the centrosomal area are shown on the right. (G) Control, n = 144; CHIR99021, n = 131; (H) Control, n = 136; CHIR99021, n = 127. Scale bar: 10 µm. P values are based on Student t test. The data underlying the graphs and blots in this figure can be found in the S2 Data and S1 Raw Images files. (S4_Fig.TIF) [file pbio.3003369.s004.tif]

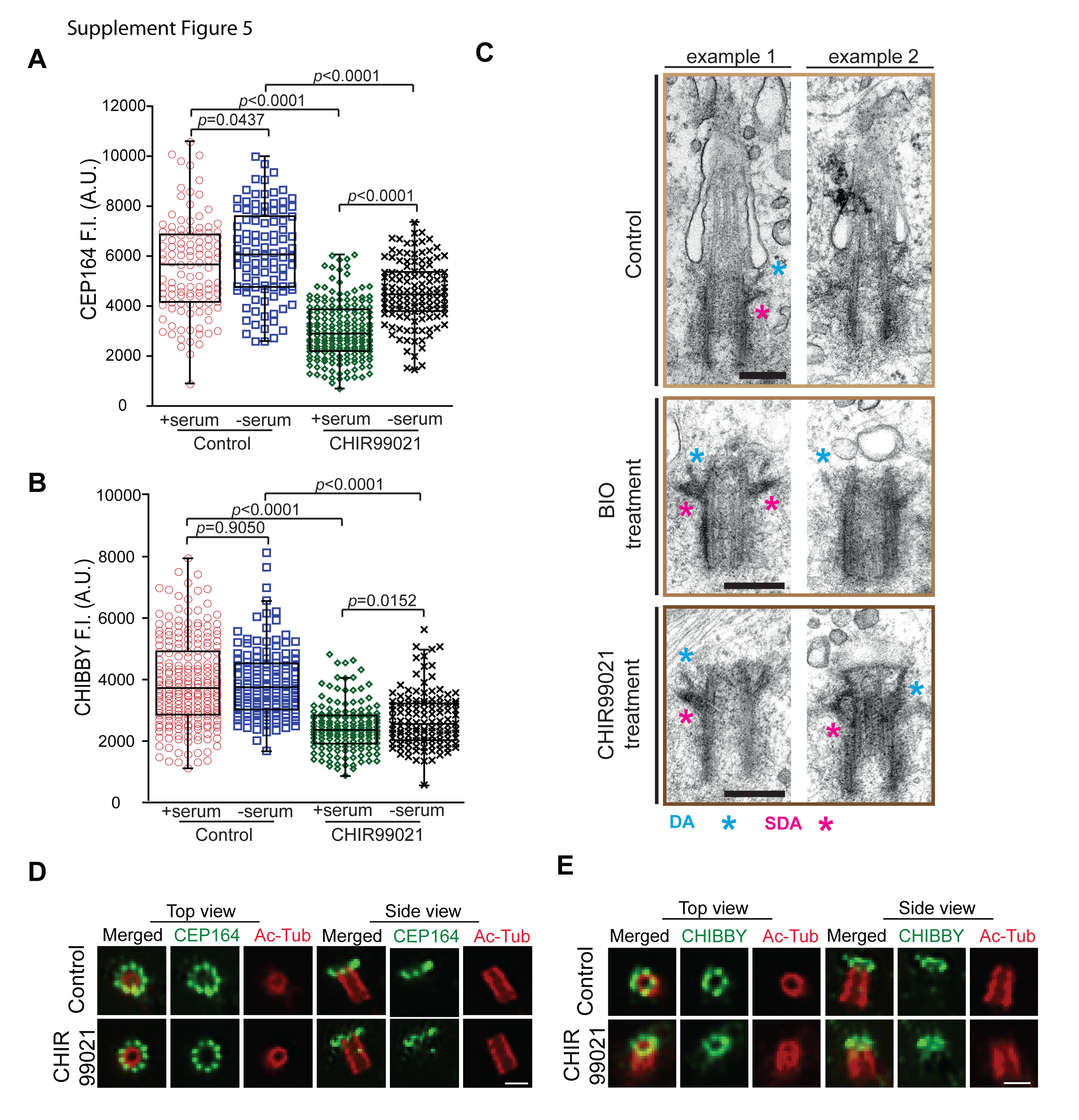

Supplement: S5 Fig — (A, B) RPE1 cells were treated with DMSO (control) or CHIR99021 for 16 h in the presence or absence of serum as depicted. Cells were stained for CEP164 (A) and CHIBBY (B). γ-tubulin served as a centriolar marker. The graph shows the fluorescence intensity of the corresponding proteins at centrosomes from three independent experiments. (A) Control, +serum, n = 118; Control, −serum, n = 101; CHIR99021, +serum, n = 187; CHIR99021, −serum, n = 125; (B) Control, +serum, n = 228; Control, −serum, n = 146; CHIR99021, +serum, n = 156; CHIR99021, −serum, n = 126. P values are based on Student t test. (C) Electron micrographs showing cross-sections of centrioles in RPE1 cells treated for 16 h with DMSO (control) or with the GSK3 inhibitors BIO and CHIR99021, as depicted. Two representative images per condition are shown. DA, distal appendages (blue asterisk), SDA, subdistal appendages (red asterisk). Scale bar: 200 nm. (D, E) U-ExM analysis of RPE1 cells treated with DMSO (control) or CHIR99021 for 16 h and stained for CEP164 (D) and CHIBBY (E). Acetylated tubulin antibodies were used to label centrioles. Representative images show enlarged top and side views of the centrosomal area. Scale bar: 250 nm. The data underlying the graphs in this figure can be found in the S2 Data file. (S5_Fig.TIF) [file pbio.3003369.s005.tif]

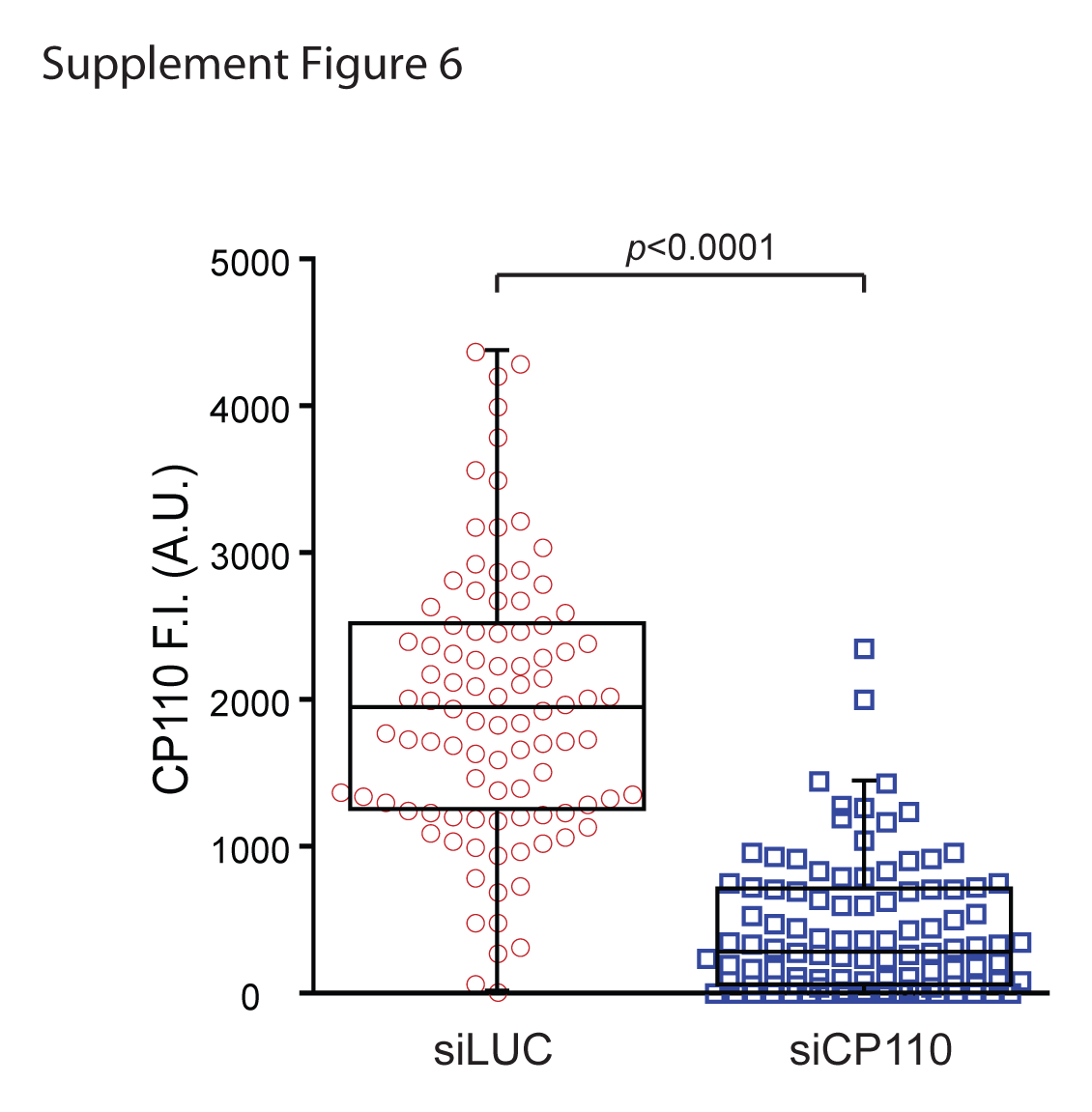

Supplement: S6 Fig — Quantification of CP110 centrosomal levels of the experiment shown in Fig 4D and 4E for RPE1 cells treated with control (siLUC) or CP110-siRNA (siCP110). Antibodies against CP110, ODF2 and acetylated tubulin were used. The graph shows the fluorescence intensity of CP110 at centrioles from three independent experiments. siLUC, n = 101; siCP110, n = 109. P values are based on Student t test. The data underlying the graphs in this figure can be found in the S2 Data file. (S6_Fig.TIF) [file pbio.3003369.s006.tif]
