# Supplementary material for: The interplay between Wnt and mTOR signaling modulates ciliogenesis in human retinal epithelial cells
Source: PLoS Biol. 2025 Sep 2;23(9):e3003369. doi: 10.1371/journal.pbio.3003369 (PMC12416842; doi:10.1371/journal.pbio.3003369)
Supplement: S1 Raw Images — The PDF file shows the corresponding uncropped membranes to each figure panel, as indicated. (S1_Raw_Images.PDF) [file pbio.3003369.s011.pdf]

Raw images for western blots

Please note that membranes shown in Figures 1D, 4G, S1A, S1B, S2B, S3C and S4A were cut longitudinally into strips for simultaneous staining with different antibodies. The stripes were reassembled for detection, and the relevant antibodies are indicated.

Figure 1D

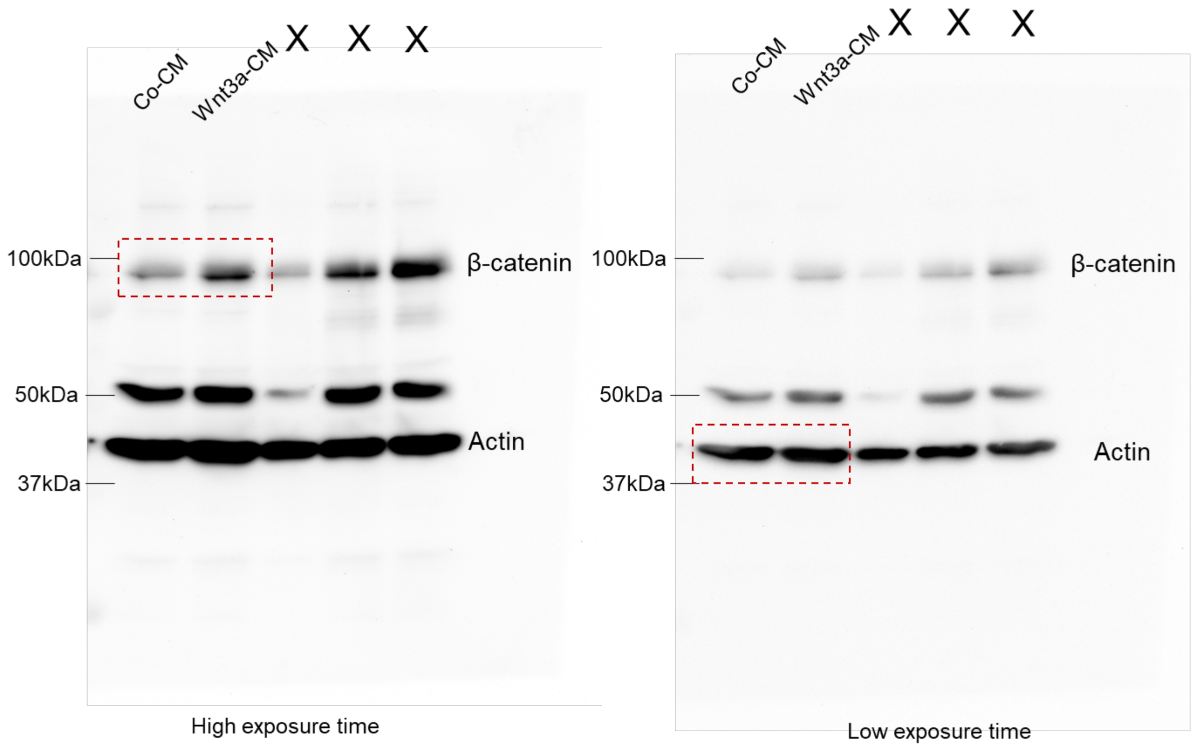

Figure 2B

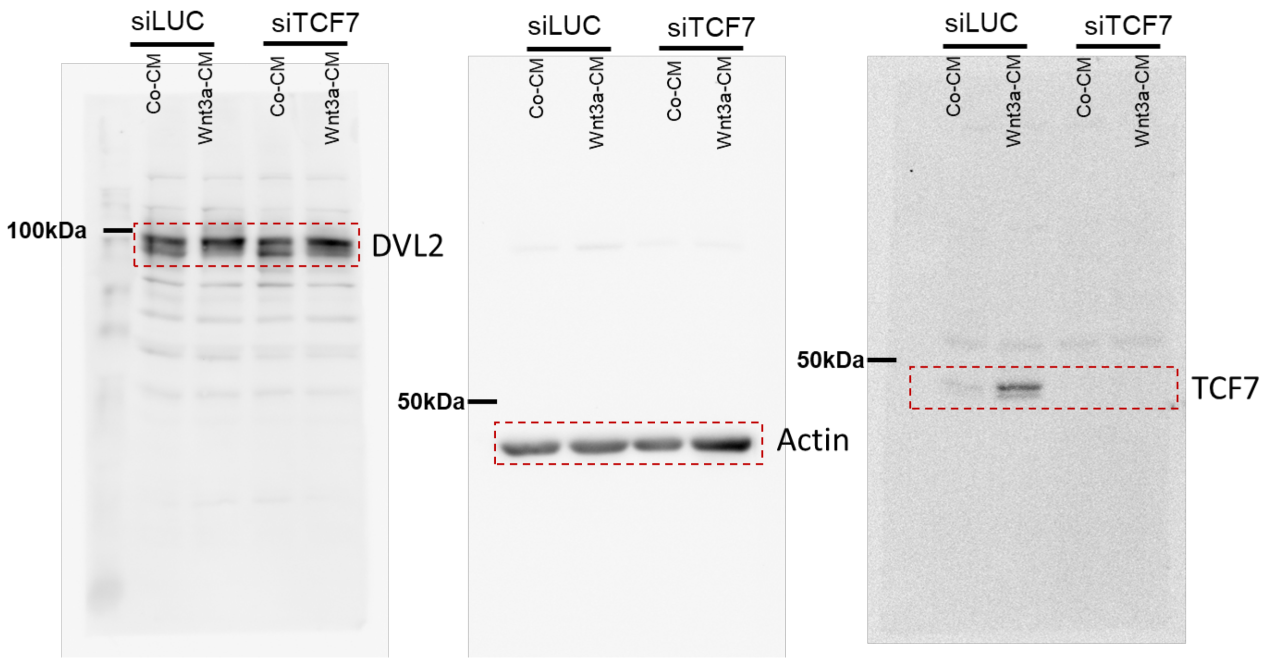
siLUC
siTCF7
Co-CM
Wnt3a-CM
Co-CM
Wnt3a-CM
100kDa
DVL2
siLUC
siTCF7
Co-CM
Wnt3a-CM
Co-CM
Wnt3a-CM
50kDa
Actin
siLUC
siTCF7
Co-CM
Wnt3a-CM
Co-CM
Wnt3a-CM
50kDa
TCF7

Figure 4G

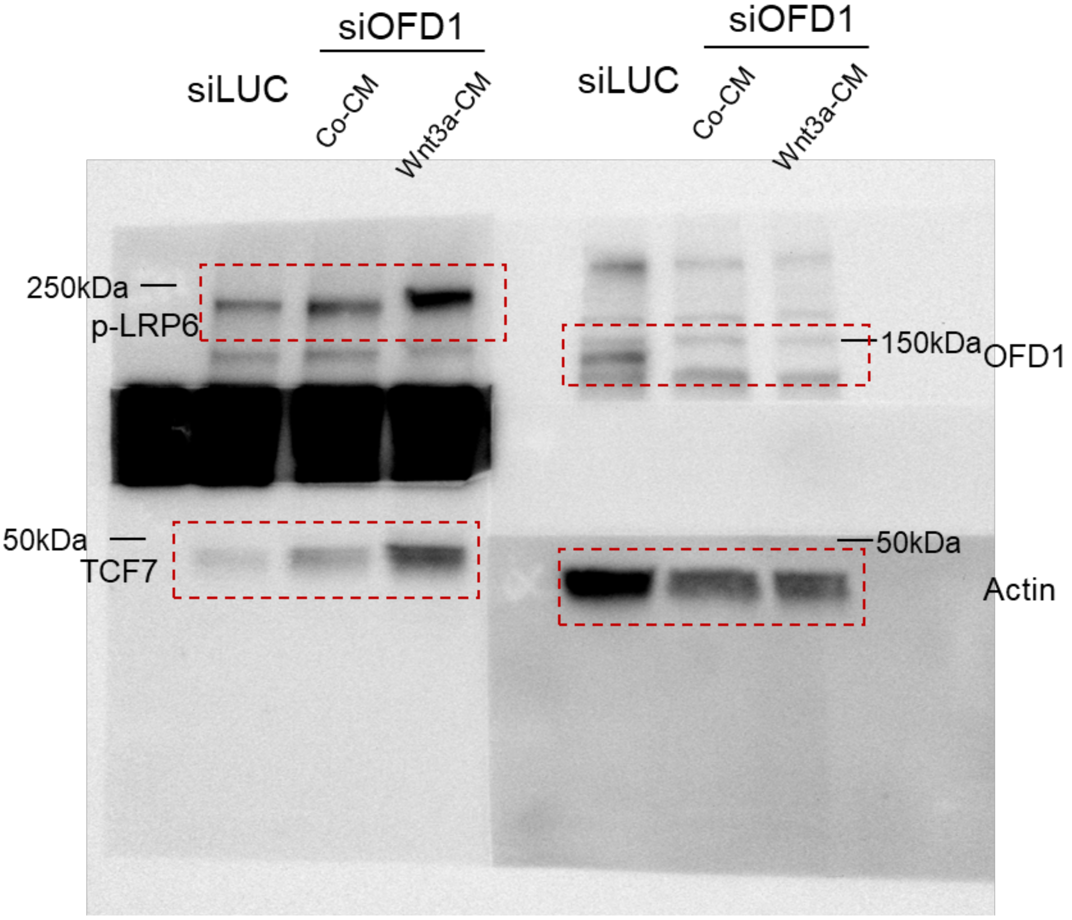

siLUC
siOFD1
Co-CM
Wnt3a-CM
siLUC
siOFD1
Co-CM
Wnt3a-CM
250kDa
p-LRP6
50kDa
TCF7
150kDa
OFD1
50kDa
Actin

Figure 5C

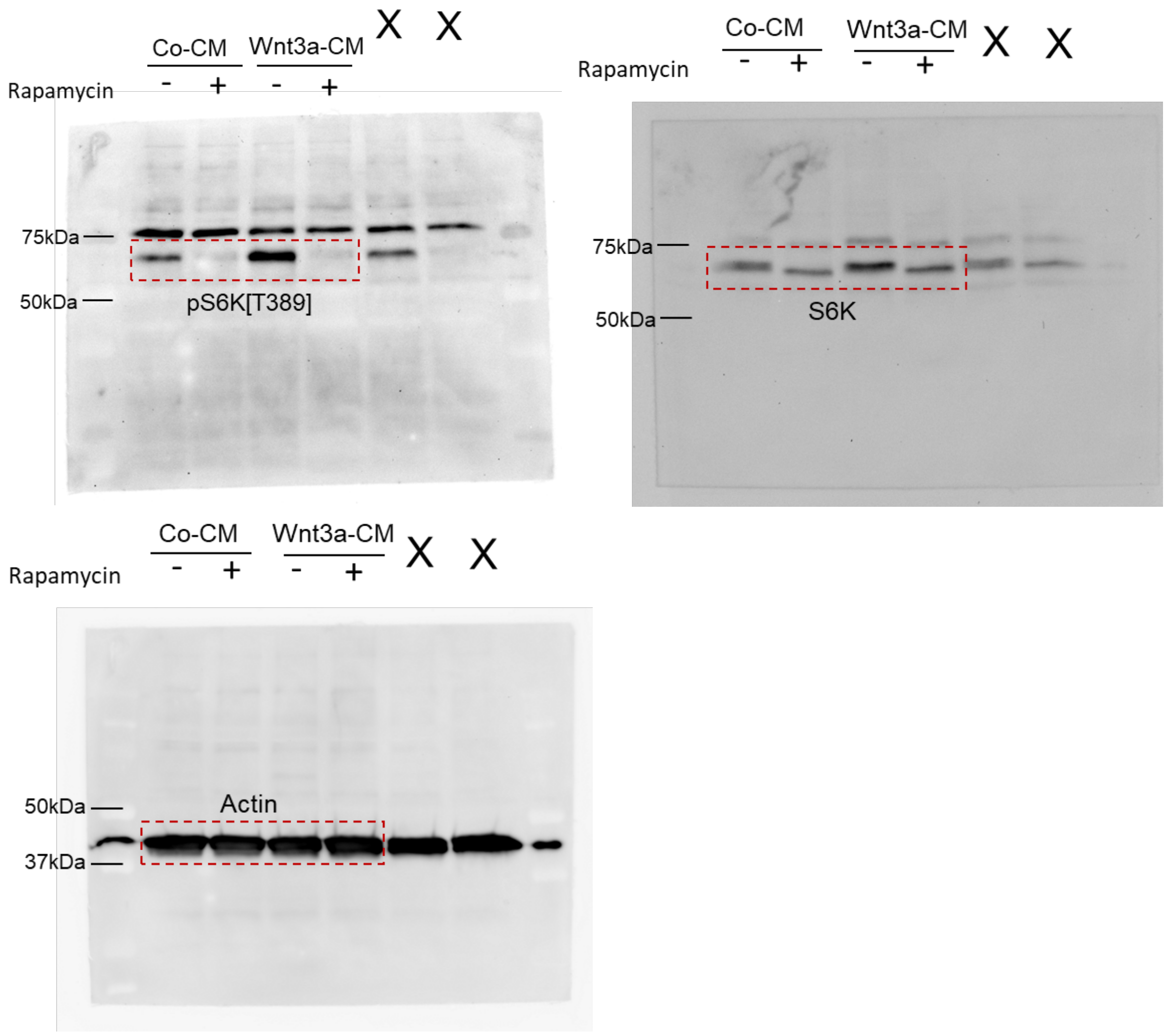
Co-CM
Wnt3a-CM
X X
Rapamycin
- + - +
75kDa
50kDa
pS6K[T389]
Co-CM
Wnt3a-CM
X X
Rapamycin
- + - +
75kDa
50kDa
S6K
Co-CM
Wnt3a-CM
X X
Rapamycin
- + - +
50kDa
37kDa
Actin

Figure S1A

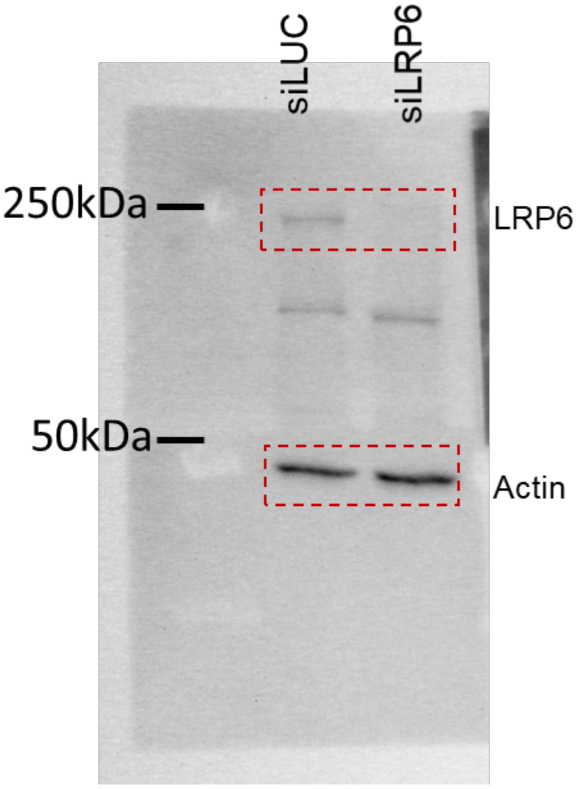

Figure S1B

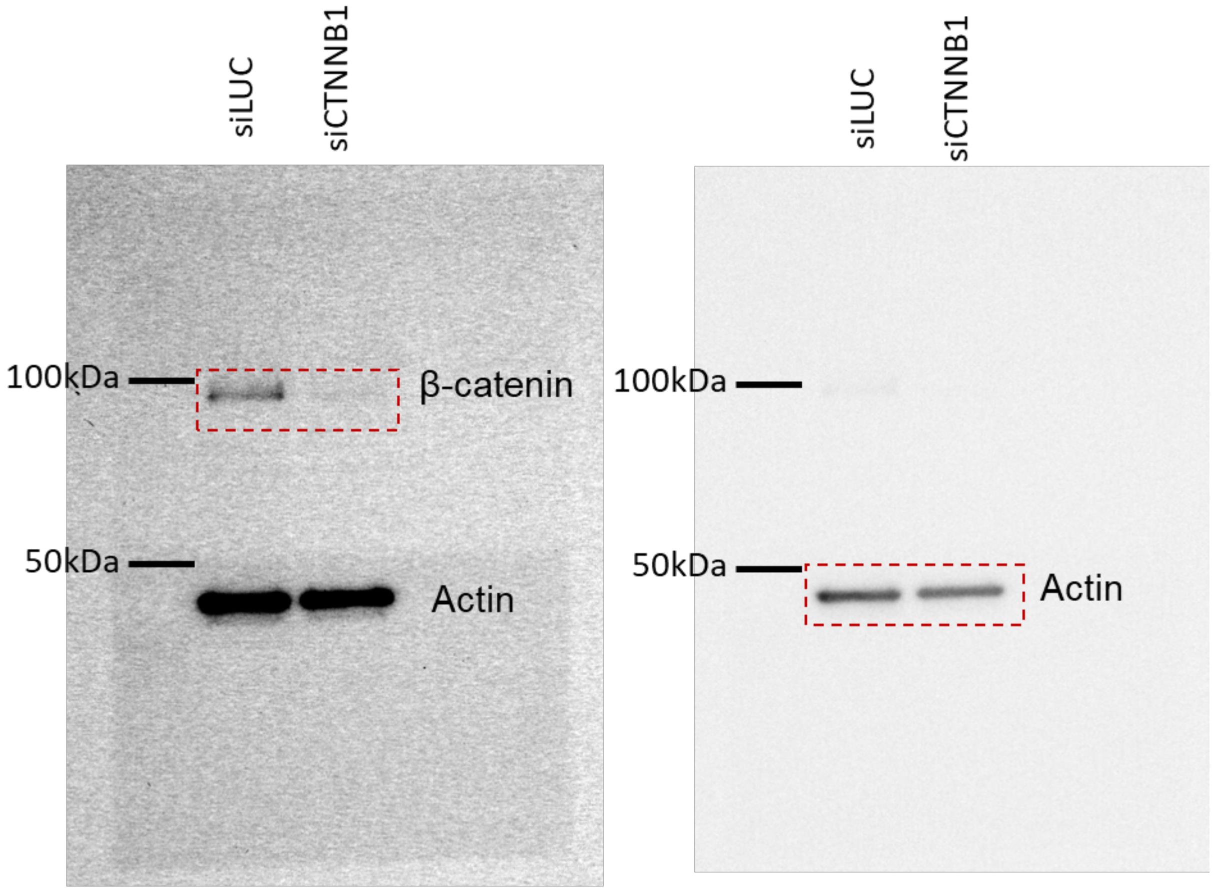

High exposure time

Low exposure time

Figure S2B

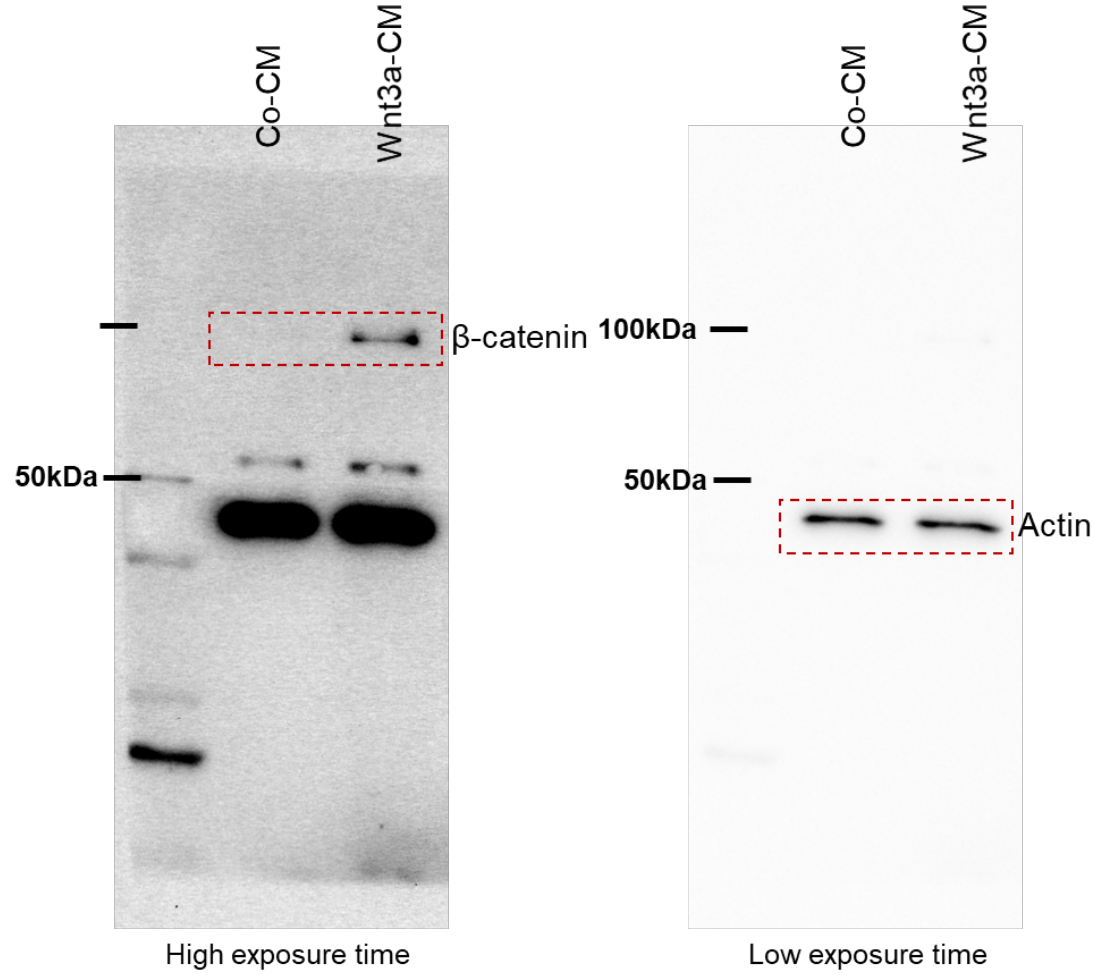
Co-CM
Wnt3a-CM
β-catenin
50kDa
High exposure time
Co-CM
Wnt3a-CM
100kDa
50kDa
Actin
Low exposure time

Figure S3C – Western blots used for quantifications, in addition to those shown in Figure 2B.

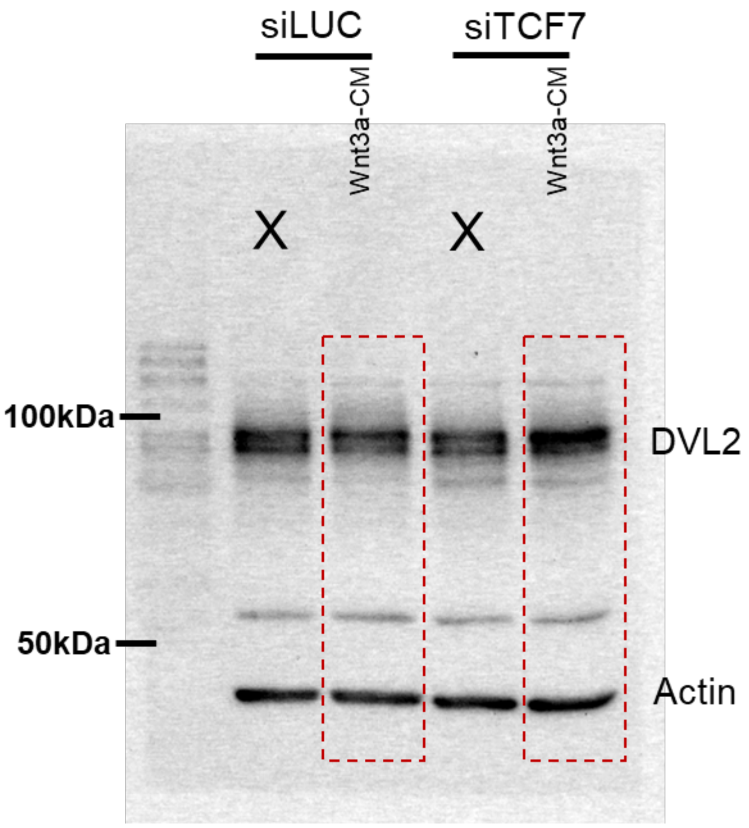

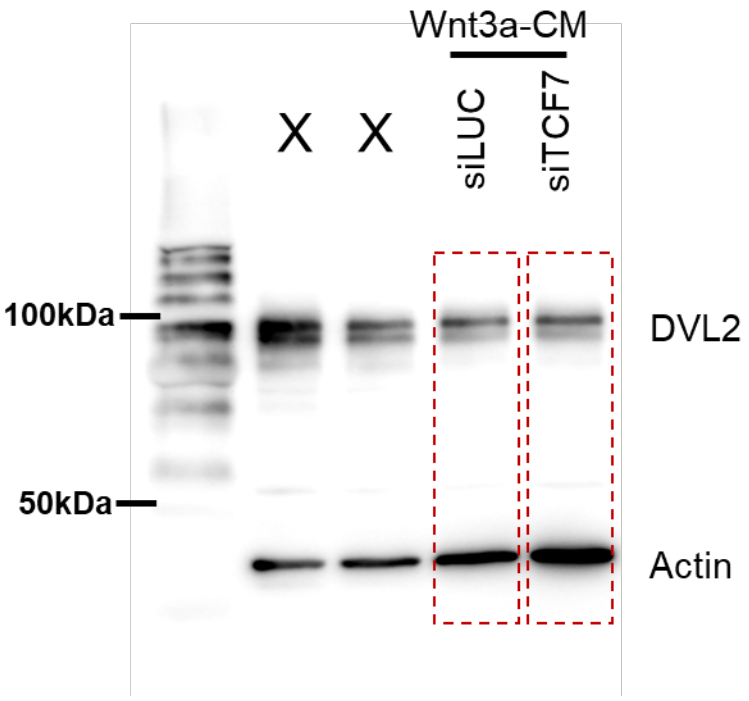

Figure S4A

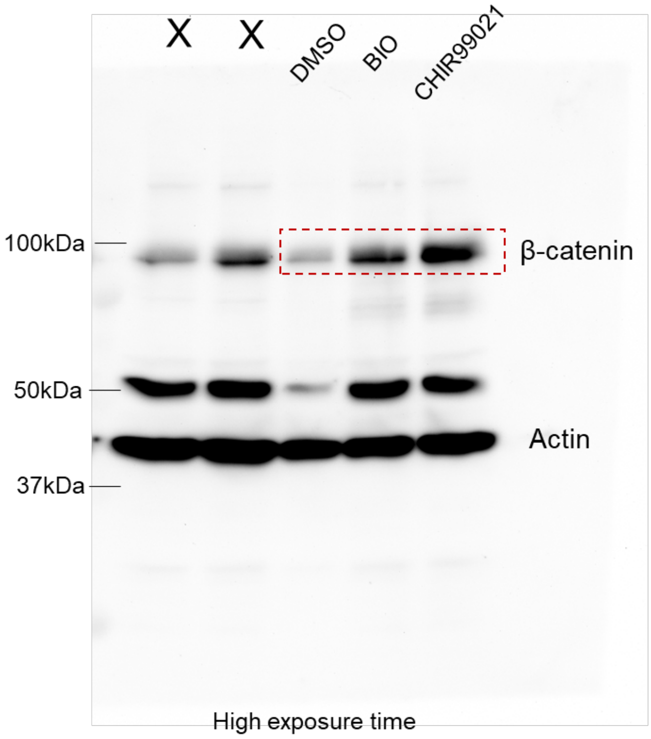

X
X
DMSO
BIO
CHIR99021
100kDa
β-catenin
50kDa
Actin
37kDa
High exposure time

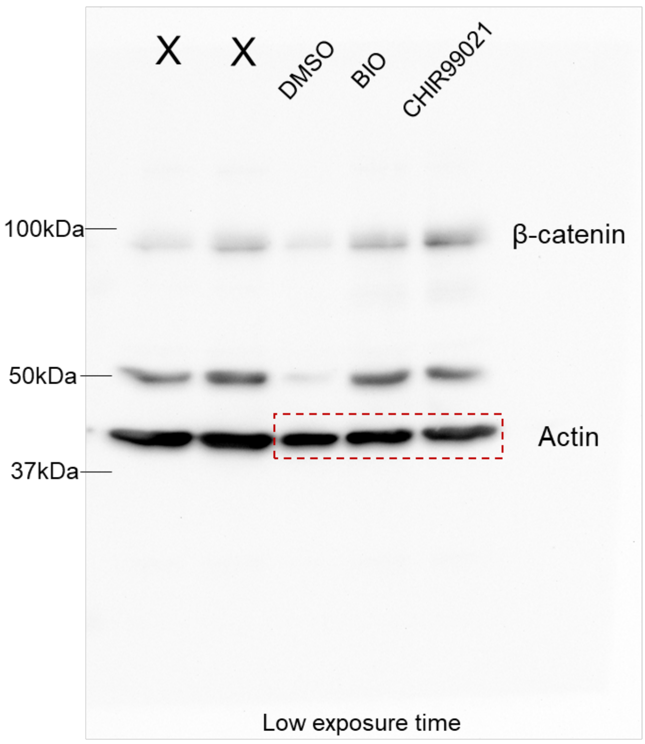

X
X
DMSO
BIO
CHIR99021
100kDa
β-catenin
50kDa
Actin
37kDa
Low exposure time
