# Supplementary material for: The interplay between Wnt and mTOR signaling modulates ciliogenesis in human retinal epithelial cells
Source: PLoS Biol. 2025 Sep 2;23(9):e3003369. doi: 10.1371/journal.pbio.3003369 (PMC12416842; doi:10.1371/journal.pbio.3003369)
Supplement: Attachment — Submitted filename: PBIOLOGY-D-25-00097R1_Response_reviewers.docx [file pbio.3003369.s014.docx]

We would like to thank both reviewers for their enthusiasm for the topic and for their constructive comments on the manuscript.

We have now addressed all points raised by the reviewers, as detailed below.

Reviewers' comments

**Rev. 1:**

The authors of this manuscript explore a highly important topic. While it has received significant attention in recent years, many previous studies have been disjointed and not carefully conducted. They show many different lines of evidence that high basal levels of Wnt signaling negatively affect ciliogenesis, and also begin to explore the mechanisms behind this. In particular they could show that early steps of ciliogenesis are blocked under high Wnt activity via satellite-bound OFD1 not being efficiently removed. In the final section of the results the authors show evidence to suggest that increased mTOR activity contributes to cilia loss under high Wnt baseline activation.

Compared to most Cilia WNT studies, experimental design has been carefully considered and shows that timing and cell line critically important. They also show that the observed effect is also seen in other cell lines.

The title is misleading. I would be careful with the wording that this work has conclusions for human RPE, since the RPE1 cells (we assume they are refereeing to the hTERT RPE1 cell line) are far removed from retinal pigment epithelial cells.

We have now modified the title to “Hyperactivation of Wnt/LRP6 signaling delays ciliogenesis in hTERT-RPE1 cells” to avoid any potential misunderstanding and additionally accommodate the suggestion from reviewer 2.

In general, better quantification of Immunos, WB and cilia lenth.

Since there are a variety of different cells lines being used. It would be very helpful to state clearly on the figures which cell line is being used when.

We have included the quantifications as detailed in the specific comments below and have specified the cell lines other than RPE1 in figures S2E and S2F.

**Specific Comments:**

Since most of the non-ciliated cells still show a small extension of ARL13, I think one needs to be careful to call these non-ciliated. Its imperative that all cilia are also measured in terms of length.

We thank the reviewer for pointing out the ARL13b signal in non-ciliated cells in Fig. 1. We realized that we had not mentioned in the manuscript that the antibody used in our original experiments (rabbit anti-ARL13b, Proteintech, batch from 2019) produces a non-specific centriolar signal. To avoid confusion, we have now replaced the representative images. Importantly, the analysis using a different batch of this rabbit anti-ARL13b antibody, which does not show the non-specific centriolar signal, confirmed our results. We now show representative images of cells stained with a new batch of rabbit anti-ARL13b (Fig. 1E).

We have measured the length of remaining cilia in cells treated with Wnt3a-CM and the Wnt agonist surrogate. We observed no significant difference compared to control samples (Fig. S3A and S3B).

Figure 1 A and B: No Results to show downregulation of these genes. Please provide evidence tos how levels of down regulation ideally on gene and protein level.

We have now included Western blot images in Fig. S1A and S1B to show the levels of LRP6 and β-catenin downregulation corresponding to Fig. 1A and 1B.

Figure 1 E: good example of figure that really needs to include quantification.

Figure 1E has been moved to 1F, and quantifications have been added.

In Figure 1 and 2 Length of cilia not just presence of cilia also important. Why not include this?

Cilia length measurements are now shown in the following figures: S1C (siLRP6), S1D (siCTNNB1), S3A (Wnt3a-CM), S3B (Wnt surrogate), and 2D (siTCF7).

Figure 3a: Why is difference between control and Wnt-3a getting smaller over time? Is Wnt induction by wnt-3a is decreasing over time. Maybe the authors can check Beta Catenin expression for other time points as they did for 16h.

The levels of β-catenin remain increased in Wnt3a-CM compared to Co-CM after 48 h of treatment (now shown in Fig. S2B). In addition, the analysis of Wnt activity using the 7xTGC reporter indicates that the percentage of GFP-positive cells (representing active Wnt signaling) remains high throughout the time course (see figure below for 60 h of treatment with Wnt3a-CM).

**Figure legend:** Images of RPE1 7xTGC cells treated with Co-CM and Wnt3a-CM under serum starved condition for 60 h.The increase in GFP signal reflects Wnt activation. Scale bar: 10 µm.

Sup. fig 1H: Nice to show Wnt surrogate affect, but from these comparisons it looks as if Wnt surrogate induces proliferation (due to more cells).

The analysis of 1H was performed in the presence of serum and we did not determine the proliferation rate of cells, as ciliogenesis assays were conducted in serum-free conditions. Although the cells grew unevenly in some areas, we did not observe an increased overall cell number upon visual inspection of the samples. To better represent the data, we have now replaced the images in S1H to show more evenly distributed cells.

Figure 2B: Quantification of this figure would be really important. Actin seems thicker for siTCF7 wnt3a-cm. So its difficult to say that TCF7 has no effect on hyperphosphorylated forms of DVL2.

For figure 2B, we have now measured the signal intensity of phospho-DVL2 relative to actin in control and siRNA-TCF7 depleted cells treated with Wnt3a-CM in three independent experiments. No significant difference was observed, supporting the conclusion that TCF7 does not affect DVL2 hyperphosphorylation under our experimental conditions. This quantification is now included in Figure S3 (panel C).

Figure 3 G-F-H: For completions sake, would it be possible to include Wnt3a for Cep164 and Chibby and CHIR for ODF2.

We have now included the quantifications for Cep164/Wnt3a (Fig. S4C), Chibby/Wnt3a (Fig. S4E) and ODF2/CHIR (Fig. S4G).

Figure 4 A: Typo MotheR centriole

This has been corrected. Thank you.

Fig. 2 E Supplement: What is happening with Cep83? The P value looks significant 0.0087. Is the number wrong? In the text they state that this is not significant?

We have re-checked the numbers and confirm that the p-values are correct. We observed a significant increase in centrosomal Cep83 levels following Wnt3a and CHIR treatment (Fig. S4F and S4H). The text has been revised accordingly to note this increase in protein levels at the centrosome (page 11).

Figure 4D: Can you quantify downregulation of CP110.

The quantification of CP110 downregulation is now included in Fig. S6, based on immunofluorescence analysis using anti-CP110 antibodies to measure centrosomal CP110 levels.

Final Model is not a good summary of the findings and difficult to interpret: Wnt induction decreases cilia number and when Wnt is active, GSK3 is inhibited. However, the authors also show that GSK3 inhibition increases ciliation, therefore based on this model (as depicted) Wnt should also increase ciliation.

We have now simplified the model (fig. 5F) to illustrate that basal Wnt activity promotes ciliogenesis, whereas hyperactive Wnt signaling delays it. Although Wnt activation inhibits GSK3, our data show that GSK3 inhibition with BIO or CHIR reduces ciliogenesis in most cells (Fig. 3A), consistent with the delay observed upon Wnt hyperactivation with Wnt3a-CM. Due to the toxicity of prolonged GSK3 inhibitor treatment, we could not determine whether ciliogenesis was delayed (rather than completely blocked), as observed with extended Wnt3a-CM treatment.

____________

**Rev. 2:**

This manuscript by Yuan and colleagues investigates the impact of Wnt/LRP6 signaling on ciliogenesis in RPE1 cells. The authors primarily employ Wnt3a-conditioned media, GSK3 and mTOR inhibition, depletion of Wnt pathway components, and centriolar/cilia components, to explore the underlying mechanisms. Overall, the results are compelling, and the observed defects in ciliogenesis are convincingly demonstrated. The authors propose that Wnt activation impairs the removal of the cilia inhibitory protein OFD1, and that mTOR inhibition can rescue cilia formation under Wnt-activated conditions.

However, I find the proposed mechanism somewhat unclear, and several points require clarification prior to publication:

1: As discussed by the authors, GSK3 inhibition (but not Wnt3a treatment) reduces the levels of distal appendage proteins CEP164 and Chibby at mother centrioles, implying a structural defect. It would be valuable to examine centriole ultrastructure under GSK3 inhibition using electron microscopy. Although the loss of Chibby is less pronounced in Wnt3a-treated cells, there still appears to be a reduction. It would strengthen the study to use super-resolution microscopy to assess whether the spatial organization of CEP164 and Chibby is preserved or not under Wnt3a treatment.

We have now analyzed centriole ultrastructure using electron microscopy (EM) and expansion microscopy (ExM). Appendages were detected by EM and no spatial disorganization of CEP164 or Chibby was observed by ExM. Therefore, we conclude that alterations in the levels of appendage components do not cause structural changes. This data has now been included in Fig. S5C to S5E.

2: Can the authors measure CEP164 and Chibby levels over a time course prior to ciliogenesis, with and without CHIR99021 treatment? Specifically, do these proteins increase during serum starvation, or are their levels stable and then reduced upon GSK3 inhibition? This would provide insight into whether GSK3 inhibition triggers active degradation of distal appendage components.

We have now compared centrosomal levels of Cep164 and Chibby in serum-fed (+ serum) and serum-starved (– serum) cells, with and without CHIR (Fig. S5). In control samples (without CHIR), centrosomal Cep164, but not Chibby, levels increased upon serum-starvation compared to serum-fed cells (Fig. S5A and S5B). These data are in agreement with our previous publication showing that centrosomal Cep164 levels increase in preparation for ciliogenesis (Schmidt et al., JCB 2012).

If GSK3 promoted the degradation of Cep164 and Chibby, CHIR treatment would be expected to increase their levels. However, this was not the case: CHIR treatment reduced centrosomal levels of both Cep164 and Chibby, regardless of serum conditions (Fig. S5A and S5B). Therefore, we conclude that the effect of CHIR on centrosomal Cep164 and Chibby is independent of cilia induction and not consistent with GSK3-mediated protein degradation.

3: For all experiments, it is unclear whether the observed impairment in ciliogenesis represents a complete block or a delay. A brief clarification in the text would help readers better interpret the results.

We have now revised the text throughout the manuscript for clarity. Based on our data, we propose that Wnt hyperactivation causes a delay in ciliogenesis rather than a complete block.

4: Figure 1, there are noticeable differences in the percentage of ciliated cells across control experiments. Could the authors explain this variability, as it may influence statistical comparisons ?

In our hands, intermediate durations of serum starvation (e.g. 16- 18 h) were more susceptible to variation compared to prolonged starvation (e.g. 48 h), with factors like cell passage number and initial seeding density partly contributing to this variability. In Figure 1 (A, B, D, G and H), cells were serum-starved for 16-18 h, resulting in an average ciliation rate of approximately 50%. To ensure statistical consistency, all experiments were repeated at least three times using the same initial cell number and cells from the same passage. We observed minimal variation between these biological replicates.

5: A brief introduction to TCF7 and its relevance in this context would be helpful for readers unfamiliar with its role in Wnt signaling.

We have now revised the text to introduce TCF7 (page 9).

6: For panels 3B-D, please use consistent color coding for protein staining to facilitate comparison.

We have now adjusted the colors in Figure 3B-D for consistency.

7: The authors state, "Collectively, the data indicate that early steps of ciliogenesis are blocked under high Wnt activity." Is the process entirely blocked, or is it delayed? Please clarify.

We propose that ciliogenesis is delayed rather than completely blocked. To avoid confusion, we have now revised the text throughout the manuscript.

8: I may have missed this data, but would it be possible to quantify ODF2 intensity under CHIR99021 treatment?

Quantification for ODF2 centrosomal levels under CHIR treatment is now shown in Figure S4G.

9: Figure 4 : In the CP110 siRNA experiments, did the authors observe microtubule elongation phenotypes, even in the absence of full ciliogenesis? This phenotype is often independent of cilium formation.

Under our conditions of CP110 siRNA depletion, over-elongated centrioles were not observed in either cycling or serum-starved cells.

10: In the OFD1 knockdown experiment, the percentage of ciliated cells appears low in both control and treated conditions. Could this reflect an early time point, consistently with the fact that Wnt signaling delay rather than completely block ciliogenesis (and what is the result at later time point)? If delay is the major phenotype, the authors might consider adjusting the title to reflect this nuance—for example, by using the term 'delayed ciliogenesis'

As discussed in points #4 and #7, we believe that variations in the controls can be attributed to differences in early ciliation dynamics. To ensure consistency and to avoid confusion, we have revised the text to state that Wnt hyperactivation delays, rather than completely blocks, ciliogenesis. Accordingly, we have revised the title to “Hyperactivation of Wnt/LRP6 signaling delays ciliogenesis in hTERT-RPE1 cells”. We hope that this title now better reflects the major findings of our manuscript.
